# Performance of Polygenic Scores for Predicting Phobic Anxiety

**DOI:** 10.1371/journal.pone.0080326

**Published:** 2013-11-20

**Authors:** Stefan Walter, M. Maria Glymour, Karestan Koenen, Liming Liang, Eric J. Tchetgen Tchetgen, Marilyn Cornelis, Shun-Chiao Chang, Eric Rimm, Ichiro Kawachi, Laura D. Kubzansky

**Affiliations:** 1 Department of Social and Behavioral Sciences, Harvard School of Public Health, Boston, Massachusetts, United States of America; 2 Department of Epidemiology and Biostatistics, University of California San Francisco, San Francisco, California, United States of America; 3 Department of Epidemiology, Mailman School of Public Health, New York, New York, United States of America; 4 Department of Epidemiology, Harvard School of Public Health, Boston, Massachusetts, United States of America; 5 Department of Biostatistics, Harvard School of Public Health, Boston, Massachusetts, United States of America; 6 Department of Nutrition, Harvard School of Public Health, Boston, Massachusetts, United States of America; Wayne State University, United States of America

## Abstract

**Context:**

Anxiety disorders are common, with a lifetime prevalence of 20% in the U.S., and are responsible for substantial burdens of disability, missed work days and health care utilization. To date, no causal genetic variants have been identified for anxiety, anxiety disorders, or related traits.

**Objective:**

To investigate whether a phobic anxiety symptom score was associated with 3 alternative polygenic risk scores, derived from external genome-wide association studies of anxiety, an internally estimated agnostic polygenic score, or previously identified candidate genes.

**Design:**

Longitudinal follow-up study. Using linear and logistic regression we investigated whether phobic anxiety was associated with polygenic risk scores derived from internal, leave-one out genome-wide association studies, from 31 candidate genes, and from out-of-sample genome-wide association weights previously shown to predict depression and anxiety in another cohort.

**Setting and Participants:**

Study participants (n = 11,127) were individuals from the Nurses' Health Study and Health Professionals Follow-up Study.

**Main Outcome Measure:**

Anxiety symptoms were assessed via the 8-item phobic anxiety scale of the Crown Crisp Index at two time points, from which a continuous phenotype score was derived.

**Results:**

We found no genome-wide significant associations with phobic anxiety. Phobic anxiety was also not associated with a polygenic risk score derived from the genome-wide association study beta weights using liberal p-value thresholds; with a previously published genome-wide polygenic score; or with a candidate gene risk score based on 31 genes previously hypothesized to predict anxiety.

**Conclusion:**

There is a substantial gap between twin-study heritability estimates of anxiety disorders ranging between 20–40% and heritability explained by genome-wide association results. New approaches such as improved genome imputations, application of gene expression and biological pathways information, and incorporating social or environmental modifiers of genetic risks may be necessary to identify significant genetic predictors of anxiety.

## Introduction

Anxiety disorders, the most commonly occurring psychiatric disorders in the United States, account for a substantial burden of disability, increased health care utilization, and high absenteeism from work [1], [2]. Twin studies typically suggest heritability of anxiety disorders between 20–40% [3], [4], and up to 50–55% for traits proposed as endophenotypes (e.g., neuroticism, behavioral inhibition) [5]. However, these twin study heritability estimates do not reveal anything about the genetic architecture of anxiety. For example, the heritability could be largely attributable to a small number of high risk alleles or a large number of low risk alleles [4], [6], [7], [8], [9].

To date, no causal genetic variants have been identified for anxiety, anxiety disorders, or related traits. Only a handful of small (n's range from 200–2,235) genome-wide association studies (GWAS) of anxiety disorders and neuroticism have been published [10], [11], [12], [13], and efforts to replicate findings from these studies have failed [12], [14], [15], [16]. Furthermore, findings from candidate gene studies have not been confirmed by GWAS. It may be that publication bias and the “winner's curse” lead to overly optimistic effect estimates in the discovery stage of gene-disease association studies. GWAS and candidate gene studies have considered multiple forms of anxiety, including panic disorder, phobic anxiety and neuroticism. However, thus far no conclusive evidence has linked a specific genetic marker with any form of anxiety, nor has evidence suggested that any one form of anxiety disorder has a stronger genetic basis than any other. Several hypotheses for the absence of confirmed loci have been posited, including the possibility that the heritability of anxiety is attributable to a large number of alleles each with effect sizes too small to detect in GWAS [17]. Evidence supporting a polygenic basis of anxiety was reported in a recent paper by Demirkan *et al.*, in which ∼2% of anxiety was predicted in independent samples using genome-wide polygenic risk scores derived from a GWAS of depression [18].

Genome-wide, pathway non-specific, or “hypothesis-free”, polygenic risk scores are constructed based on the associations for a large number of alleles meeting nominal p-value significance criteria, even while assuming that many or even most of these alleles are false positives. While a typical GWAS tests millions of single nucleotide polymorphisms (SNPs) for association with the phenotype of interest and applies very conservative alpha criteria (*e.g.*, 5×10^−8^) for statistical significance, polygenic risk scores incorporate information from SNPs that are not genome-wide significant. If polygenic risk scores explain substantial variance in anxiety phenotypes, it would provide indirect evidence for the common disease – common variant hypothesis; common alleles, each with a small effect on disease, largely explain the heritability of complex polygenic diseases [18], [19].

To test the hypothesis that heritability of phobic anxiety can be explained by common SNPs, we considered the variation in phobic anxiety that can be explained using GWAS-derived polygenic risk scores in genotyped sub-samples of women from the Nurses' Health Study (NHS) and men from the Health Professionals Follow-up Study (HPFS). We also assessed a polygenic risk score based on 31 candidate genes identified *a priori* from published literature. Finally, we attempted to replicate the finding from Demirkan and colleagues by creating a polygenic score (developed from the GWAS of depression in their sample) using weights identical to those in the original study [18].

## Materials and Methods

### Ethics Statement

The NHS and HPFS were approved by the Human Subjects Committee of Brigham and Women's Hospital, Boston, MA. All participants in this study provided written informed consent.

### Population

All data are drawn from 7 nested case-control GWAS within the NHS and HPFS cohorts.

#### Nurses' Health Study (NHS)

The NHS was established in 1976 when 121,700 female registered nurses aged 30–55 years and residing in 11 large U.S. states completed a mailed questionnaire on medical history and lifestyle characteristics [20]. Participants have been followed with repeated questionnaires on lifestyle and health every 2 years. Blood was collected from 32,826 participants between 1989 and 1990. DNA was extracted from white blood cells using the QIAmp™ (QIAGEN Inc., Chatsworth, CA) blood protocol and all samples were processed in the same laboratory. Genome-wide scans were obtained from 4 independent GWAS of the cohort, initially designed to examine type 2 diabetes (T2D), coronary heart disease (CHD), breast cancer (BrCa) and kidney stone (KS) disease. Both cases and controls were included in the current analyses because the link between any given genotype or gene score and anxiety was expected to be independent of disease status. For the NHS T2D GWAS, 3286 participants were genotyped with controls defined as women free of diabetes at the time of diagnosis of the case, and matched on year of birth, month of blood collection, and fasting status. These matched-pairs were subsequently broken because not all subjects gave informed consent to post their data on dbGaP. For the NHS CHD set 1146 participants were included. CHD cases were matched on age, smoking, and month of blood draw to controls (1∶2) randomly selected from women who provided blood samples and did not have CHD on the date of diagnosis of the case. For the NHS BrCa study (n = 2287) cases and controls were limited to post-menopausal women. Controls were women without breast cancer and matched 1∶1 with cases by age and post-menopausal hormone use at blood draw. In the NHS KS study (n = 504), cases were women with a history of kidney stones, and controls randomly selected from among women with no history of cancer or cardiovascular disease and who met age eligibility requirements for every case (1∶0.5).

Considering quality control (QC) and available information on phobic anxiety a total of 7002 genotyped participants were available from NHS (NHS T2D = 3105, NHS CHD = 1133, NHS BrCa = 2274, NHS KS = 490).

#### Health Professionals Follow-up Study (HPFS)

The HPFS was initiated in 1986 when 51,529 male health professionals between ages 40 and 75 years and residing in the U.S. completed a questionnaire on lifestyle and medical history. Participants have been followed with repeated questionnaires on lifestyle and health every 2 years.

Between 1993 and 1996, a blood sample was requested from all active participants and collected from 18,225 men [21]. DNA was extracted from white blood cells using the QIAmp™ (QIAGEN Inc., Chatsworth, CA) blood protocol; all samples were processed in the same laboratory. Genome-wide scans were obtained from 3 independent GWAS of the cohort, initially designed to examine T2D, CHD, and KS disease. Both cases and controls were included for analysis. In the HPFS T2D (n = 2487), CHD (n = 1313), and KS (n = 553) participants were genotyped following the same design and method for selecting cases and controls as used in the NHS parallel sub-studies. In total, 4125 HPFS participants (T2D n = 2279, CHD n = 1294, KS n = 552) were included, after restrictions based on QC and missing information on phobic anxiety.

### Genotyping and Imputation

Exact genotyping, QC, and imputation protocols varied by sample set (Tables S1 and S2). DNA samples that did not meet at least 90% (NHS) or 95%(HPFS) completion threshold and SNPs with low call rates <90% (NHS) or ≤95% (HPFS) were dropped. Principal components analyses were conducted to exclude self-reported white individuals that had substantial similarity to non-European reference samples [22]. Each study imputed up to 2.5 million autosomal SNPs with NCBI build 36 of Phase II HapMap CEU data (release 22) as the reference panel using MACH [11], [19]. Imputation results summarized as allele dosage were used for analysis. dbGaP accession numbers for the publicly funded genotyping are: NHS T2D (phs000091.v2.p1), NHS BrCa (phs000147.v1.p1), , HPFS T2D (phs000091.v2.p1), NHS/HPFS KS (phs000460.v1.p1). The genotyping for NHS/HPFS CHD was supported by Merck and are not in dbGaP.

### Phenotype

Anxiety symptoms were assessed using the phobic anxiety scale of the Crown Crisp Experimental Index (CCI). The scale includes 8 questions about fear of crowds, heights, enclosed spaces, and going out alone, and worrying; items have two or three response options. Items with 3 response options were scored as no (0), moderate (1), or high (2); symptom level items with 2 response options were coded as no (0), or high (2) symptom level. We summed across items, resulting in an overall score ranging from 0 to 16 with higher scores indicating higher levels of phobic anxiety. For those with missing items, the total score was divided by the fraction of questions answered and then rounded to the nearest whole number, so the possible range of total scores was consistent across all individuals.

This scale discriminates individuals with diagnosable anxiety disorders from healthy individuals, correlates reasonably with other measures from the Middlesex Hospital Questionnaire (free-floating anxiety *ρ* = 0.48, obsessional *ρ* = 0.45, somatic *ρ* = 0.42,depressive *ρ* = 0.27) [23], has high intra-class correlation in monozygotic twins (0.60) and high heritability (0.64) [24], and is associated with heart disease in both men and women [25], [26], [27]. To reduce the impact of measurement error or short term fluctuations in CCI, we averaged two CCI assessments (NHS: 1988, 2004; HPFS: 1988, 2000). For cohort members who provided only one CCI score, we used that measure. The distribution of the CCI score stratified by case-control status is reported in Table S3.

### Analyses

#### Genome Wide Association Analysis

Genome-wide association analyses were conducted separately for each of the 7 sample sets. We related dosage genotype across 2.5 million SNPs to the continuous phobic anxiety score using linear regression under an additive genetic model. Even though we analyzed unrelated individuals, we additionally adjusted for the top three or four eigenvectors to address residual population stratification. Fixed-effects meta-analysis with GWAMA was used to combine the results of the 7 cohorts [28], [29].

#### Agnostic (Hypothesis-Free) Genome Wide Polygenic Risk Score Profile

To evaluate the predictive value of a combination of SNPs across studies, the meta-analyzed results from the genome-wide association analyses were restricted to 1.54 million SNPs imputed with R^2^>95% across all study samples and further restricted to a set of 94,657 independent SNPs using the PLINK pruning procedure. Briefly, specifying a window of 200 SNPs, the corresponding LD between each pair of SNPs was calculated and one of the pair of SNPs was removed if the LD was greater than 0.25. Subsequently the window was shifted forward by 5 SNPs and the procedure repeated.

We estimated polygenic risk scores following prior similar work [18], [19]. This method entails 3 steps: 1) estimating beta weights for each SNP based on a GWAS in a discovery sample; 2) restricting to SNPs with p-values below a pre-specified threshold (we considered alternative thresholds ranging from 0.00001 to 0.5); 3) calculating a polygenic risk score for each individual in a target sample as the sum of risk alleles from the previously selected list of SNPs, with each SNP weighted by the beta estimate from the discovery sample.

Genome-wide data for both NHS and HPFS were collected in separate nested case control studies, resulting in 7 distinct samples in our analyses. For each individual in each sample, the risk score was calculated using the SNPs and beta weights derived from the other 6 samples as the meta-analyzed discovery set. The risk score for predicting phobic anxiety was calculated by multiplying the estimated beta weight by the number of risk alleles at each SNP (0, 1, 2) and summing across all SNPs in the SNP set defined by the p-value threshold from the discovery set.

We then used linear regression to quantify the association between the polygenic risk score and the phobic anxiety phenotype in each target sample. After iterating across all seven studies, always leaving out one study as the target sample, the resulting variances explained (R^2^) from the scoring procedure were meta-analyzed by weighting with the sample size of each target set.

#### Replication of Previously Established Polygenic Risk Score

Demirkan and colleagues previously used the above procedure to develop a polygenic risk score for major depressive disorder as assessed by the Composite International Diagnostic Interview [30]. They tested the score in Rotterdam Study participants (age 55 years and older) using a case-control design with 222 anxiety disorder cases (including generalized anxiety disorder, panic disorder, agoraphobia, social phobia and specific phobia, assessed with Munich Composite International Diagnostic Interview (M-CIDI). In analyses comparing these cases to 290 controls with no M-CIDI disorder and a Hospital Anxiety and Depression-Anxiety score of zero, the polygenic score explained approximately 2% of the variance. Demirkan and colleagues (personal communication) provided the precise beta weights (log odds) and p-values used in this analysis to facilitate creation of the polygenic score and replication in our cohorts. To be consistent with the Demirkan analysis which dichotomized anxiety, we defined a dichotomous phobic anxiety phenotype:scoring 4+ on the phobic anxiety scale at either assessment (Table 1), chosen based on prior work [25]. We assessed the association between the score derived by Dermirkan and colleagues and our dichotomous phenotype using logistic regression R^2^.

**Table 1:** Descriptive statistics of 11127 study participants.

	NHS (7002 females)		HPFS (4125 males)	
	Mean/Frequency	Standard Deviation/(%)	Mean/Frequency	Standard Deviation/(%)
Age at blood draw (yrs)	57.8	6.8	62.2	9.2
1^st^ Measure of Phobic Anxiety*	2.8	2.2	1.9	1.9
2^nd^ Measure of Phobic Anxiety**	2.8	2.4	1.9	1.9
Mean of 1^st^ and 2^nd^ Measurement of Phobic Anxiety	2.8	2.1	1.9	1.7
Ever Phobic Anxiety? (1^st^ or 2^nd^ Measurement ≥4)	2937	42.0	1068	25.9

* Missing Information: NHS = 175, 5.1% of participants with item non-response, 0.1% with item non-response >2 out of 8 items; HPFS = 328, 1.1% of participants with item nonresponse, 0.1% with item non-response >2 out of 8 items.

** Missing Information: NHS = 1093 (726 due to death) 4.1% of participants with item non-response, 0.2% with item non-response >2 out of 8 items; HPFS = 479 (294 due to death), 5.1% of participants with item non-response, 0.2% with item non-response >2 out of 8 items.

#### Candidate Gene Risk Score

In addition to using GWAS to identify likely candidate SNPs, we selected the following 31 candidates genes identified in prior literature [5], [6], [10], [31], [32]. *ADORA2A, ADRB1, ANO2, ARRDC4, BDNF, CALCOCO1, CCK, CCKBR, CLU, COMT, CRH, CRHR1, GAD1, GPC6, HTR1A, HTR2A, HTR3A, MAPT, MDGA2, NKAIN2, OXT, PDE4D, PKP1, PLEKHG1, PLXNA2, RGS2, SDK2, SLC1A1, SLC6A3, SLC6A4, TPH2*. These candidate genes were matched to the imputed SNPs by chromosomal position +/−20 kb using the USCS genome browser via http://genome.ucsc.edu/. The 7984 SNPs that matched to the genes were restricted to those that were well imputed with R^2^>95% across all studies (n = 4887) and further pruned to 370 independent SNPs with the same methodology as described above with each gene being represented by at least one SNP. A candidate gene risk score was derived using these candidate gene SNPs with weights derived from the meta-analysis described above and the same p-value thresholds used for testing the GWAS-derived polygenic score.

#### Proportion of Phenotypic Variance Explained

To address the capacity of the common SNPs genotyped on the different platforms to explain phobic anxiety, we estimated the phenotypic variance explained in each case-control sample following the approach suggested by Yang et al. [33] using the standard Genome-wide Complex Trait Analysis (GCTA) protocol. We present the average of the GCTA estimates, calculated with weighting by sample size; these results should be interpreted cautiously because they combine GCTA information from different platforms.

## Results

Of the 11,127 participants with information on anxiety and genotype, 63% were female (Table 1). The first and second measurement of the CCI phobic anxiety index were correlated at Pearson r = 0.59.

**Figure 1:**
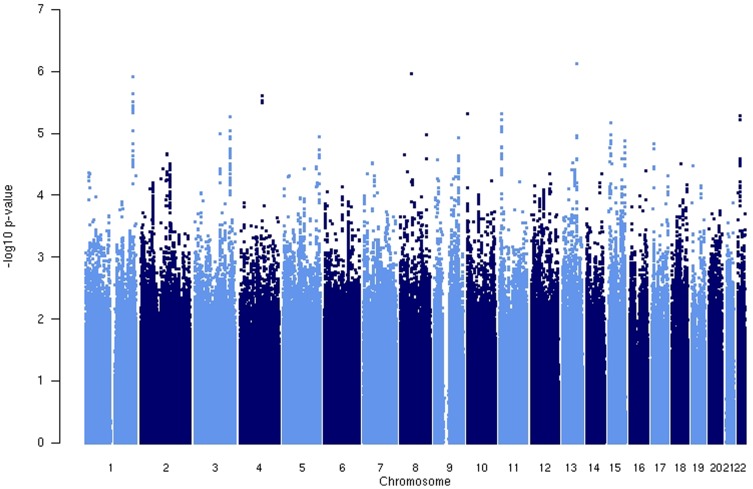
Genome wide association plot (Manhattan plot), phobic anxiety in the Nurses' Health Study and the Health Professionals Follow-up Study (n = 11,127).

In the genome-wide meta-analysis of all seven data sets (Figure 1), no SNP reached genome-wide significance (i.e. significant at p<5×10^−8^) but 10 independent signals passed the suggestive threshold of p<1×10^−5^ (Table 2). The SNP with the strongest association was rs4911015 (p = 7.38×10^−7^) on chromosome 13.

**Table 2:** Most statistically significant Single Nucleotide Polymorphisms based on GWAS Results from Meta-Analysis of 7 Cohorts (p<1×10−5).

SNP	Chr	Position	A1	A2	Frequency (A1)	Effect	Standard Error	P-value	Effect direction	n	Heterogeneity (i2)	P-value (Hetero-geneity)	# Support SNPs*	Closest Gene (<50 kb)
rs4911015	13	84985734	A	T	0.87	−0.20	0.04	7.38E-07	−−−+−−−	11125	0.00	0.82	9	
rs426691	8	55294178	C	T	0.61	−0.14	0.03	1.08E-06	−−−−−−+	11126	0.46	0.07	2	
rs17825699	1	230254964	T	G	0.76	0.16	0.03	1.20E-06	+++++++	11125	0.00	0.61	0	DISC1
rs9728264	1	230291141	G	A	0.42	0.12	0.03	2.31E-06	+++++++	11125	0.11	0.88	18	DISC1
rs789651	1	230760474	A	C	0.96	−0.31	0.07	4.47E-06	−−−−−−−	11125	0.00	0.59	11	SIPA1L2
rs12787660	11	11109384	G	A	0.54	0.12	0.03	4.84E-06	+++++++	11126	0.00	0.21	7	
rs10794718	10	1132750	T	C	0.47	−0.12	0.03	4.85E-06	−+−−−−−	11127	0.55	0.24	0	KIAA0982
rs3788398	22	25160478	G	A	0.95	0.26	0.06	5.17E-06	+++++++	11124	0.00	0.32	19	ASPHD2
rs12636740	3	174527418	G	A	0.75	−0.15	0.03	5.48E-06	−−−−−−−	11126	0.00	0.66	31	
rs1258724	15	30848487	T	C	0.74	−0.13	0.03	6.82E-06	−−−−−−−	11127	0.00	0.97	11	FMN1

Chr = Chromosome, A1 = coded allele, A2 = non-coded allele.

* Number of SNPs within 250 kb of indicator SNP, linkage disequilibrium threshold of R2≥0.5, and p for association <0.01.

In meta-analyzed results with independent target samples, the derived agnostic polygenic risk score was never associated with phobic anxiety at nominally significant (p<.05) thresholds for any of the tests. The variance explained (Table 3) was negligible (maximum 0.01%) regardless of which p-value threshold was used to select SNPs for inclusion in the risk score calculation. These results were consistent across several modifications of the inclusion criteria, including relaxing the restriction of 95% imputation quality and using the complete set of correlated SNPs to derive the risk scores, or additionally adjusting for case status in the respective case-control studies (results not shown).

**Table 3:** Meta-Analyzed R^2^ for the Association between the Polygenic Risk Score and Phobic Anxiety, across Seven Target Samples.

P-value threshold for SNP inclusion in polygenic risk score	R^2^ for polygenic risk score predicting phobic anxiety (variance explained)*	P-value for the genome-wide polygenic risk score	R^2^ for candidate gene risk score predicting phobic anxiety (variance explained)*	P-value for the genome-wide polygenic risk score	NagelkerkeR^2^ for Demirkan risk score predicting phobic anxiety∧	P value of association of the associated score
0.00001	0.000	0.65	–	–	–	–
0.0001	0.000	0.77	–	–	0.001	0.63
0.001	0.000	0.77	–	–	0.001	0.48
0.01	0.001	0.23	0.001	0.12	0.001	0.26
0.1	0.001	0.14	0.001	0.19	0.002	0.82
0.2	0.001	0.11	0.001	0.10	0.001	0.42
0.3	0.001	0.10	0.001	0.35	0.001	0.16
0.4	0.001	0.30	0.001	0.38	0.001	0.07
0.5	0.001	0.10	0.001	0.28	0.001	0.12

* R^2^ estimates based on linear regression of phobic anxiety on polygenic risk score in each target sample, based on polygenic risk score estimates from GWAS of the other 6 samples.

∧ NagelkerkeR^2^ values from logistic regressions using Demirkan score to predict dichotomized phobic anxiety phenotype.All models adjusted for eigenvectors.

We next applied the Demirkan weights to construct the depression-derived polygenic risk score in our sample. This score did not predict the dichotomized phobic anxiety phenotype in our sample, even with weights and p-value thresholds identical to those applied by Demirkan et al. (Table 3); nor did it predict the continuous phobic anxiety score or a more extreme dichotomous outcome using the highest scoring decile of the CCI distribution as the cut-off value (results not shown). To facilitate comparison with the results derived from the internal score and to address concerns regarding the combined use of cases and controls in this analysis, we provide additional regression results showing no association between the depression-derived polygenic risk score and continuous the continuous phobic anxiety score in Table S4.

The candidate gene risk score, based on SNPs in local neighborhoods of 31 candidate genes, was also unrelated to the phobic anxiety phenotype (Table 3).

Exploratory GCTA analyses revealed an estimated heritability explained by all common SNPs ranging from 0% for 31%, averaging to 17% across the 7 different study samples (Table S5).

We have developed a set of policies and guidelines to accommodate independent review upon request.

## Discussion

In a GWAS of a large sample of men and women of European descent, we found no genome-wide significant associations with phobic anxiety. Phobic anxiety was also not associated with a polygenic risk score derived from the GWAS beta weights using liberal p-value thresholds; with a previously published genome-wide polygenic score; or with a candidate gene risk score based on 31 genes previously hypothesized to predict anxiety.

Genome-wide, pathway non-specific, hypothesis-free polygenic risk scores such as those used in this study potentially capture small elevations in risk associated with SNPs that would not meet conventional genome-wide significance thresholds. This approach may thus ameliorate the “false negative” problem in typical GWAS, but this advantage comes at the costs of adding noise to the prediction because many “false positives” are included in the score calculation. To address this inflation of “false positives” in the score we tested a polygenic risk score restricted to SNPs located in the neighborhood of candidate genes incorporating prior knowledge about the genetics and biology of anxiety.

Genome-wide polygenic risk scores have been informative for traits like schizophrenia, bipolar disorder, coronary heart disease and type II diabetes [19], [34] though less informative for cancer [35]. When substantial phenotypic variance can be explained by genome-wide polygenic risk scores, despite few or no individual SNPs that meet the genome-wide significance threshold, it supports the common disease-common variant hypothesis. Such a finding would suggest genetic risk is distributed across many independent loci with small effects.

Our null findings cannot be considered to disconfirm the common disease common variant hypothesis for phobic anxiety, however. Common genetic variants with very small effects may exist but be undetectable in this analysis because of lack of statistical power. This study had 80% power to detect a single SNP associated with 0.16 SD unit change of the continuous anxiety phenotype if such a SNP had a minor allele frequency of 5%; equivalently, we had power to detect a single SNP explaining 0.34% of the variance. Assuming a 20% chip heritability as suggested by GCTA (Table S5) and prior twin studies, an average training data set of 9 000 individuals and a target data set of 2000 individuals, the power of the polygenic risk score in our analysis was 80% to detect a nominally significant association if the genetic risk was distributed across 1% of the risk markers considered [36]. Even if the genetic risk was distributed across all 94 657 SNPs considered in the polygenic risk score, our analyses still had 75% power to detect an association at a p-value threshold of 0.5 (Table S6).

There are several possible explanations for our inability to replicate the prior association between a genome-wide polygenic risk score and anxiety [18]. Anxiety is a defining feature of several related disorders. Though specific anxiety symptoms are common to most of the disorders, variation in heritability estimates may be partly attributed to differences across anxiety disorders. For example, panic disorders, as defined by repeated and unexpected panic attacks, can be distinguished phenotypically from phobic disorders, exemplified by the fear of developing panic-like symptoms and avoidance behavior with respect to specific object or situation [6]. The genetics of phobic anxiety, the phenotype in our study, may differ from the genetics of the HADS-A, generalized anxiety, measure used in Demirkan *et al.* Nonetheless, because all anxiety disorders aggregate in families attributed to shared genetic risk [3], and because data from a population-based twin registry suggests that the genetic components of anxiety are shared across different anxiety disorders [4], we would have expected at least a partial replication of their findings [5], [6], [32]. It is also possible that, given the divergence of the demographic and social conditions of the populations in the two studies, the finding in an older Dutch community sample do not generalize to our occupationally-based U.S. cohorts.

Nonetheless our results are consistent with most research on anxiety genetics. Of three GWAS of neuroticism [11], [12], [13], [16], an endophenotype of anxiety, none of the findings have been replicated. Likewise, candidate genes studies have not established replicable risk genes [17]. Together, these results suggest that understanding the genetics of anxiety may require very large sample sizes and also a broader analytic approach. It seems increasingly likely that heritability is not conferred mainly through SNPs but through other genetic or epigenetic modifications, such as deletions, inversions, translocation and differential gene regulation as recent findings regarding anxiety and related mental disorders suggest that miRNA, rare alleles, copy number variations, and epigenetic modifications may play key roles in shaping the phenotypes [14], [15], [37], [38], [39], [40].

Substantial gene-environment interactions may also obscure the relevant genetic risk factors, and inflate heritability estimates from twin studies. If genotype-phenotype associations are heterogeneous across environmental contexts, the associations may be absent or diluted in certain environments when this gene-environment interaction is not recognized and explicitly modeled. We did not directly assess this, but the continuing difficulty of identifying specific risk alleles despite established heritability supports the possibility of important environmental modifiers of genetic risk.

This study has important limitations. As with all GWAS, sample size is a limitation; a larger sample size improves the statistical power to detect signals and in the case of polygenic risk scores reduces statistical noise in the different prediction models, particularly when applying lower p-value thresholds. We used a continuous phenotype measure to improve statistical power, although results were similar with a dichotomized measure (data not shown). But we did not have information on medication usage or other treatments for anxiety, and the phenotype may therefore not be manifest in successfully treated individuals; to the extent that anxiety treatments are successful, this could reduce power. In addition, polygenic risk scores such as those applied in this study assume an additive genetic model without interactions which might not reflect the underlying genetic architecture of the trait. Moreover, findings in these cohorts, recruited from health professionals in the U.S., may not be generalizable to representative population samples. Lastly, the failed attempt to replicate the findings from Demirkan *et al.* may be explained by genetic effects specific to a particular phenotype of anxiety; this would be somewhat surprising given the evidence from twin studies suggesting a common liability across anxiety disorders [4]. Our results do not conclusively rule out the possibility of many polymorphisms each with very small effects on anxiety symptoms.

Our study was based on secondary analyses of case-control sample sets; under the null hypothesis of no genotype-phenotype association, (i.e., in the present study, the genetic risk score and anxiety ), there is no bias introduced by case-control sampling even if the phenotype of interest is a correlate of the original condition used to define case-control status [41].

Despite these limitations, our study is the largest study to date to investigate the genetics of phobic anxiety. We took advantage of repeated measures of phobic anxiety to develop a more stable phenotype. We considered the research question with three distinct approaches, each with differing strengths and limitations: GWAS, polygenic risk scores and candidate gene risk scores. Results from the three approaches were very similar in that none provided genetic predictors of phobic anxiety.

### Conclusion and Future Research Suggestions

Large scale consortia with a common definition of a symptomatic measure of anxiety symptoms, clinical anxiety, or usage of anxiety associated pharmaceuticals are needed to assess potential (additive) genetics of anxiety using the common GWAS approaches. That said, new approaches may be necessary to identify powerful genetic predictors of anxiety. Critical next steps include search for genetic or environmental modifiers of genetic effects; improved genome imputations (*e.g.* 1000 genomes); and application of information on gene expression and biological pathways to prioritize a subset of genes for closer analyses and thereby mitigate the ‘false negative challenge in genome-wide analyses. In particular, identifying social modifiers of genetic risks might improve statistical power and help explain the “missing heritability” gap between GWAS results and twin-study findings.

## Supporting Information

Table S1 **Genetic Quality Control.**
(DOCX)

Table S2 **Study-specific Genotyping, Imputation and Statistical Analysis.**
(DOCX)

Table S3 **Distribution of the Anxiety Score in different Samples of Cases and Controls.**
(DOCX)

Table S4 **Association of Continuous Polygenic Risk Score based on the Demirkan Algorithm with Anxiety Symptoms, based on Linear Regression Models.**
(DOCX)

Table S5 **Results from Genome-wide Complex Trait Analysis.**
(DOCX)

Table S6 **Power Analyses for Polygenic Score.**
(DOCX)
