# Supplementary material for: Performance of Polygenic Scores for Predicting Phobic Anxiety
Source: PLoS One. 2013 Nov 20;8(11):e80326. doi: 10.1371/journal.pone.0080326 (PMC3835914; doi:10.1371/journal.pone.0080326)
Supplement: Table S1 — Genetic Quality Control. (DOCX) [file pone.0080326.s001.docx]

**Table S1. Genetic Quality Control**

| **Study** | **Sample Quality Control** | | **Sample size included** |
| --- | --- | --- | --- |
|  | **Call rate** | **Other exclusion criteria** |  |
| NHS T2D | >98% | -sex discrepancy with genetic data from X-linked markers  -duplicates and first/second degree relatives  -ancestry outliers  -heterozygosity  -autosomal chromosome abberations  -missing phenotype information | 3,105 |
| NHS CHD | >98% | -sex discrepancy with genetic data from X-linked markers  -duplicates and first/second degree relatives  -ancestry outliers  -heterozygosity  -missing phenotype information | 1,133 |
| NHS KS | ≥95% | -duplicates and first/second degree relatives  -ancestry outliers  -missing phenotype information | 490 |
| NHS BrC | >90% | -duplicates and first/second degree relatives  -ancestry outliers  -missing phenotype information | 2,274 |
| HPFS T2D | >98% | -sex discrepancy with genetic data from X-linked markers  -duplicates and first/second degree relatives  -ancestry outliers  -heterozygosity  -autosomal chromosome abberations  -missing phenotype information | 2,279 |
| HPFS CHD | >98% | -sex discrepancy with genetic data from X-linked markers  -duplicates and first/second degree relatives  -ancestry outliers  -heterozygosity  -missing phenotype information | 1,294 |
| HPFS KS | ≥95% | -duplicates and first/second degree relatives  -ancestry outliers  -missing phenotype information | 552 |
