# Supplementary material for: Performance of Polygenic Scores for Predicting Phobic Anxiety
Source: PLoS One. 2013 Nov 20;8(11):e80326. doi: 10.1371/journal.pone.0080326 (PMC3835914; doi:10.1371/journal.pone.0080326)
Supplement: Table S2 — Study-specific Genotyping, Imputation and Statistical Analysis. (DOCX) [file pone.0080326.s002.docx]

**Table S2. Study-specific Genotyping, Imputation and Statistical Analysis**

| **Study** | **Genotyping** | | | | | **Imputation** | | | **Association Analysis** | | | |
| --- | --- | --- | --- | --- | --- | --- | --- | --- | --- | --- | --- | --- |
|  | **Platform** | **Inclusion criteria** | | | **SNPs met**  **QC criteria** | **Software** | **Inclusion criteria** | | **SNPs in**  **meta-analysis** | **λ_GC_** | **Software** | **covariates** |
|  |  | **MAF** | **Call rate** | **P HWE** |  |  | **MAF** | **Imputation quality** |  |  |  |  |
| NHS T2D | Affymetrix 6.0 | ≥2% | >98% | ≥10^-4^ | 704,409 | MACH | >2% | Rsq ≥0.5 | 2,348,981 | 1.024 | ProbABEL | top 3 eigenvectors |
| NHS CHD | Affymetrix 6.0 | ≥2% | >98% | ≥10^-4^ | 721,316 | MACH | >2% | Rsq ≥0.5 | 2,351,014 | 1.003 | ProbABEL | top 3 eigenvectors |
| NHS KS | Illumina 610Q | ≥1% | ≥95% | ≥10^-5^ | 546,344 | MACH | >2% | Rsq ≥0.5 | 2,361,579 | 0.993 | ProbABEL | top 4 eigenvectors |
| NHS BrC | Illumina 550k | ≥1% | ≥90% | - | 528,173 | MACH | >2% | Rsq ≥0.5 | 2,363,494 | 0.996 | ProbABEL | top 4 eigenvectors |
| HPFS T2D | Affymetrix 6.0 | ≥2% | >98% | ≥10^-4^ | 706,040 | MACH | >2% | Rsq ≥0.5 | 2,352,767 | 1.002 | ProbABEL | top 4 eigenvectors |
| HPFS CHD | Affymetrix 6.0 | ≥2% | >98% | ≥10^-4^ | 724,881 | MACH | >2% | Rsq ≥0.5 | 2,355,075 | 1.008 | ProbABEL | top 3 eigenvectors |
| HPFS KS | Illumina 610Q | ≥1% | ≥95% | ≥10^-5^ | 546,344 | MACH | >2% | Rsq ≥0.5 | 2,361,579 | 0.995 | ProbABEL | top 4 eigenvectors |
