# Supplementary material for: Performance of Polygenic Scores for Predicting Phobic Anxiety
Source: PLoS One. 2013 Nov 20;8(11):e80326. doi: 10.1371/journal.pone.0080326 (PMC3835914; doi:10.1371/journal.pone.0080326)
Supplement: Table S3 — Distribution of the Anxiety Score in different Samples of Cases and Controls. (DOCX) [file pone.0080326.s003.docx]

**Table S3. Distribution of the Anxiety Score in different Samples of Cases and Controls**

| *Study* | *Mean Anxiety Score in Cases (SD)* | *Mean Anxiety Score in Controls*  *(SD)* | *p-value for difference in means = 0* |
| --- | --- | --- | --- |
| *NHS* |  |  |  |
| *T2D* | *3.04 (2.38)* | *2.63 (2.07)* | *<0.001* |
| *BrCa* | *2.75 (2.24)* | *2.68 (2.34)* | *0.49* |
| *CHD* | *2.61(2.47)* | *2.75 (2.34)* | *0.37* |
| *KS* | *2.90 (2.11)* | *2.53 (1.76)* | *0.04* |
| *HPFS* |  |  |  |
| *T2D* | *1.84 (2.34)* | *1.76 (2.08)* | *0.39* |
| *CHD* | *1.65 (2.21)* | *1.78 (2.01)* | *0.33* |
| *KS* | *1.94 (1.78)* | *1.72 (1.80)* | *0.15* |
|  |  |  |  |
