# Supplementary material for: Performance of Polygenic Scores for Predicting Phobic Anxiety
Source: PLoS One. 2013 Nov 20;8(11):e80326. doi: 10.1371/journal.pone.0080326 (PMC3835914; doi:10.1371/journal.pone.0080326)
Supplement: Table S4 — Association of Continuous Polygenic Risk Score based on the Demirkan Algorithm with Anxiety Symptoms, based on Linear Regression Models. (DOCX) [file pone.0080326.s004.docx]

**Table S4. Association of Continuous Polygenic Risk Score based on the Demirkan Algorithm with Anxiety Symptoms, based on Linear Regression Models.**

| \|  \| ALL (n=11127) \|  \| CONTROLS  (n=6079) \|  \| CASES (n=5048) \|  \| \| --- \| --- \| --- \| --- \| --- \| --- \| --- \| \| P-value threshold for SNP inclusion \| R2* \| P value** \| R2 * \| P value ** \| R2 * \| P value ** \| \|  \|  \|  \|  \|  \|  \|  \| \| 0.00001 \| -- \| -- \| -- \| -- \| -- \| -- \| \| 0.0001 \| 0.001 \| 0.9 \| 0.003 \| 0.82 \| 0.001 \| 0.69 \| \| 0.001 \| 0.001 \| 0.82 \| 0.002 \| 0.67 \| 0.001 \| 0.7 \| \| 0.01 \| 0.000 \| 0.13 \| 0.000 \| 0.43 \| 0.001 \| 0.26 \| \| 0.1 \| 0.001 \| 0.45 \| 0.001 \| 0.53 \| 0.002 \| 0.57 \| \| 0.2 \| 0.001 \| 0.42 \| 0.001 \| 0.19 \| 0.002 \| 0.9 \| \| 0.3 \| 0.001 \| 0.24 \| 0.001 \| 0.06 \| 0.002 \| 0.86 \| \| 0.4 \| 0.001 \| 0.11 \| 0.001 \| 0.07 \| 0.002 \| 0.59 \| \| 0.5 \| 0.001 \| 0.13 \| 0.002 \| 0.06 \| 0.000 \| 0.66 \| \|  \|  \|  \|  \|  \|  \|  \|   * R^2^ for Demirkan risk score predicting phobic anxiety. ** P value of association of the associated genetic score. |  |
| --- | --- | --- | --- | --- | --- | --- | --- | --- | --- | --- | --- | --- | --- | --- | --- | --- | --- | --- | --- | --- | --- | --- | --- | --- | --- | --- | --- | --- | --- | --- | --- | --- | --- | --- | --- | --- | --- | --- | --- | --- | --- | --- | --- | --- | --- | --- | --- | --- | --- | --- | --- | --- | --- | --- | --- | --- | --- | --- | --- | --- | --- | --- | --- | --- | --- | --- | --- | --- | --- | --- | --- | --- | --- | --- | --- | --- | --- | --- | --- | --- | --- | --- | --- | --- | --- | --- | --- | --- | --- | --- | --- | --- |
