# Supplementary material for: Performance of Polygenic Scores for Predicting Phobic Anxiety
Source: PLoS One. 2013 Nov 20;8(11):e80326. doi: 10.1371/journal.pone.0080326 (PMC3835914; doi:10.1371/journal.pone.0080326)
Supplement: Table S5 — Results from Genome-wide Complex Trait Analysis. (DOCX) [file pone.0080326.s005.docx]

**Table S5. Results from Genome-wide Complex Trait Analysis*^,^****

|  | NHS | | | | HPFS | | |
| --- | --- | --- | --- | --- | --- | --- | --- |
|  | T2D | BrCa | CHD | KS | T2D | CHD | KS |
|  |  |  |  |  |  |  |  |
| n | 3266 | 2274 | 1133 | 494 | 2444 | 1295 | 552 |
|  |  |  |  |  |  |  |  |
| Chip Heritability in % (SE) | 0.21 (0.10) | 0.06 (0.13) | 0.31 (0.28) | 0.00 (0.66) | 0.18 (0.10) | 0.20 (0.22) | 0.05 (0.34) |
|  |  |  |  |  |  |  |  |
| p | 0.01 | 0.30 | 0.14 | 0.5 | 0.01 | 0.14 | 0.44 |
|  |  |  |  |  |  |  |  |

* Please note that we considered the all the available samples for this analyze, without reducing duplicates excluded in prior analyses.

** The results rely on the genotyped results from different platforms preventing a straightforward comparison of the results.
