# Supplementary material for: Performance of Polygenic Scores for Predicting Phobic Anxiety
Source: PLoS One. 2013 Nov 20;8(11):e80326. doi: 10.1371/journal.pone.0080326 (PMC3835914; doi:10.1371/journal.pone.0080326)
Supplement: Table S6 — Power Analyses for Polygenic Score. (DOCX) [file pone.0080326.s006.docx]

**Table S6. Power Analyses for Polygenic Score**

|  | Fraction of SNPs associated with phenotype (1-null fraction) | | |
| --- | --- | --- | --- |
| P-value Threshold Used to Calculate Scores | 100% | 25% | 1% |
| 0.00001 | 5.0% | 5.1% | 99.3% |
| 0.0001 | 5.2% | 5.3% | >99.9% |
| 0.001 | 6.2% | 7.0% | >99.9% |
| 0.01 | 12.9% | 15.8% | 99.5% |
| 0.1 | 44.8% | 48.9% | 91.6% |
| 0.2 | 60.1% | 62.3% | 86.3% |
| 0.3 | 68.1% | 69.5% | 83.1% |
| 0.4 | 72.5% | 73.3% | 81.1% |
| 0.5 | 75.2% | 75.6% | 79.8% |

Threshold: P values threshold used to construct polygenic score.
Nullfraction: Percentage of SNPs with null effect on phobic anxiety.
Power: probability of rejecting the null hypothesis when the null hypothesis is false.
